# Orofacial Tumours at the Oral and Maxillofacial Unit of Komfo Anokye Teaching Hospital: A 2‐Year Retrospective Study

**DOI:** 10.1002/puh2.70240

**Published:** 2026-04-21

**Authors:** Gift Obiageri Maduagwu, Nana Bempong Owusu‐Ankomah, Nana Atuahene Oti, Afia Boko Ampe Asare, John Billy Owusu Quarshie, Elijah Kwegyir Johnson, Kwame Adu Okyere Boadu

**Affiliations:** ^1^ Komfo Anokye Teaching Hospital Kumasi Ghana; ^2^ KNUST Hospital Kumasi Ghana; ^3^ Sunyani Teaching Hospital Sunyani Ghana; ^4^ Kumasi South Hospital Kumasi Ghana

**Keywords:** benign, malignant, neoplasm, orofacial

## Abstract

**Background and Aims:**

Orofacial tumours represent a significant health challenge in Ghana impacting functions and quality of life. Despite their importance, there is a dearth in literature on the epidemiological patterns of these neoplasms in Ghana. This study aims to bridge this knowledge gap by investigating their characteristics at a major teaching hospital in Ghana.

**Methods:**

A retrospective, cross‐sectional study was conducted on 204 patients with consecutive sampling. Data were retrieved from the Lightwave Health Information Management System. Ethical approval was obtained from the Institutional Review Board of Komfo Anokye Teaching Hospital. Descriptive statistics were used to summarize the data. Chi‐square tests were performed to assess associations between age, gender and tumour type.

**Results:**

The study included 204 patients with a mean age of 40.24 ± 22.268 years. There was a slight female predominance (51.0%) with a male‐to‐female ratio of 1:1.04. Benign tumours (59.8%) were more prevalent than the malignant ones (40.2%). The highest incidence of orofacial tumours was observed in the 5th decade of life (16.7%). The mandible was the commonest site of tumour occurrence (18%), followed by the tongue (12%). A significant association was found between age and tumour type (*p* < 0.001), with benign tumours more common in younger age groups (peak in 31–40 years, 18.9%) and malignant tumours more prevalent in older individuals (peak in 61–70 years, 24.4%).

**Conclusions:**

This study provides valuable insights into the epidemiology of orofacial tumours in Ghana, highlighting the importance of age‐specific screening and early detection strategies. The findings contribute to the understanding of orofacial tumour patterns in Ghana and underscore the need for further research to elucidate underlying factors contributing to these patterns.

## Graphical abstract

This retrospective study of 204 patients in Ghana identifies the mandible as the most common tumour site. Findings reveal a significant association between age and tumour type, with benign lesions predominating in younger adults and malignancies peaking in the elderly, emphasizing the need for age‐specific screening.

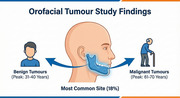

## 1 Introduction

Tumours in the orofacial region include a wide range of lesions that develop in the mouth, jaws and adjoining structures of the face. These tumours can be either benign or malignant – with varying presentations, treatment approaches and outcomes depending on their specific type and location [1, 2]. Orofacial tumours can affect chewing, speaking and swallowing. Treatment often involves complex surgical procedures, which can result in facial disfigurement [3, 4]. The presentation and treatment of orofacial tumours depend on several factors including the tumour's location, size, stage and histological type, as well as the patient's overall health and preferences. The survival rates of patients with orofacial tumours are influenced by various factors such as the tumour stage, presence of lymph node(s) or distant metastasis and complications or recurrence [1, 5].

The global prevalence of orofacial tumours varies, based on factors such as location, age and gender. Squamous cell carcinoma (SCC) is the most common type of oral cavity cancers (90%) [6, 7]. Recent literature also highlights the role of advanced imaging modalities and careful preoperative assessment to differentiate benign lesions like pleomorphic adenoma from malignant mimics, particularly when they present in atypical locations or with unusual dimensions [8]. In Ghana, research conducted indicates a high prevalence of orofacial tumours with SCC being the most frequent [9, 10, 11]. Despite the global impact of orofacial tumours, there is inadequate epidemiological data and published research on these neoplasms from sub‐Saharan Africa [9, 10, 11]. There is limited literature in Ghana to examine the distribution of tumour types diagnosed and treated. The inadequate epidemiological data on the prevalence and pattern of different histological types of orofacial tumours limit development of effective screening, diagnostic and management protocols tailored to the Ghanaian setting.

The current knowledge gap leads to delayed diagnosis and suboptimal treatment outcomes in affected patients. At Komfo Anokye Teaching Hospital (KATH), which is a major referral centre in Ghana, there is limited comprehensive analysis of the profile of orofacial tumours diagnosed and treated over the past several years. This hinders allocation of appropriate resources for diagnosis, treatment and rehabilitation services that this complex and underserved group of patients require. There is an urgent need for an in‐depth study to elucidate the frequency, demographic patterns, anatomical distribution and histopathological profile of orofacial tumours managed at the hospital.

The 2‐year retrospective study of patient records at the Oral and Maxillofacial Unit aimed to bridge this critical gap in knowledge. Findings from the study may guide formulation of protocols for early detection, multidisciplinary treatment guidelines and recommendations for resource planning to effectively manage patients with orofacial tumours in the hospital and across Ghana.

## 2 Methods

### 2.1 Study Design

This study was a descriptive retrospective study. Clinical records of patients who attended the Oral and Maxillofacial Unit of KATH from 1 January 2021 to 31 December 2023, were retrieved and analysed.

### 2.2 Profile of Study Area

This research was carried out in the Oral Health Directorate of KATH, Kumasi. It is the main referral centre for inhabitants in the Ashanti, Bono and Ahafo regions. It has a 1200‐bed capacity. The Oral Health Directorate consists of four main departments, of which the Oral and Maxillofacial Surgery Unit (OMFSU) is one [12].

### 2.3 Study Population

The study population involved all patients who presented to the OMFSU with an orofacial tumour from 1 January 2021 to 31 December 2023.

### 2.4 Sample Size Determination

The Cochran's formula was adopted in determining the required sample size: *N* = *Z* ^2^(*p*)(*q*)/*d* ^2^), where *N* is the minimum sample size required for the study, *Z* is the desired confidence level at 95% (standard value of 1.96), and *p* is the estimated proportion with attribute of interest; the prevalence of benign odontogenic tumours among patients was 15% in Ghana [11]. *d* = precision (=5%, which is expressed as 0.05); *q* = 1 − *p*. Calculation *N* = (1.96)^2^ × 0.15 × (1 − 0.15) (0.05)^2^ *N* = 195.9 ≈ 196. Therefore, the minimum sample size (*N*) required was 196.

### 2.5 Sampling Procedure

A total sampling approach was used to select patients’ data. Data that met the inclusion criteria were used for the study.

### 2.6 Operational Definition of Terms

An orofacial tumour is defined as a diverse group of neoplastic lesions—both benign and malignant—that originate from the tissues of the oral cavity and the maxillofacial region. These include tumours of epithelial origin (e.g., SCC), mesenchymal origin (e.g., sarcomas) and odontogenic origin (e.g., ameloblastoma), as well as those arising from the salivary glands and bone [13]. Operationally, a case is often defined by its ICD‐10 (International Classification of Diseases) code (e.g., C00–C06 for oral cavity, C07–C08 for salivary glands or D10–D11 for benign counterparts). Diagnosis was made on both clinical history, examinations, investigations and histopathological findings.

### 2.7 Inclusion and Exclusion Criteria

The study employed specific criteria for participant selection. Inclusion criteria encompassed all patients who presented with orofacial tumours within the study period as mentioned supra. Exclusion criteria, on the other hand, comprised patients with the following dataset missing—(i) types of tumours, (ii) tumour site and (iii) age.

### 2.8 Data Collection and Analysis

Existing medical records on the Lightwave Health Information Management System (LHIMS) in KATH for patients who presented at Oral and Maxillofacial Unit were used for data collection. The data included gender, age of first presentation at the Oral and Maxillofacial Unit, tumour type (clinical diagnosis) and treatment received. An adapted structured checklist was used to collect the data.

### 2.9 Scale Development and Measurement

The research instrument utilized a comprehensive 5‐point Likert scale, ranging from 1 (Strongly Disagree) to 5 (Strongly Agree), to assess knowledge, attitudes and practices. Scale development followed a rigorous process including extensive literature review of existing dental health questionnaires, expert panel review by dental professionals, pilot testing for comprehension and cultural appropriateness, and careful translation and back‐translation for local language versions.

### 2.10 Statistical Analysis

All statistical analyses were performed using SPSS version 27. The results were presented using a variety of methods such as tables and bar charts. Sex differences in tumour type and other measures were analysed employing quantitative statistical methods like *t*‐test.

### 2.11 Ethical Considerations

Ethical approval was obtained from the Committee on Human Research, Publications and Ethics (CHRPE/AP/367/24) of the School of Medicine and Dentistry (SMD), Kwame Nkrumah University of Science and Technology and from KATH Institutional Review Board (KATH‐IRB). The data collected were well kept under lock, ensuring confidentiality of the patient's information. The data were solely used for the intended study purpose, and identities of patients were kept anonymous.

### 2.12 Patient Consent for Publication

No written consent was obtained from patients as there was no patient‐identifiable data included in this case report/series.

## 3 Results

### 3.1 Demographic Features

**FIGURE 1:**
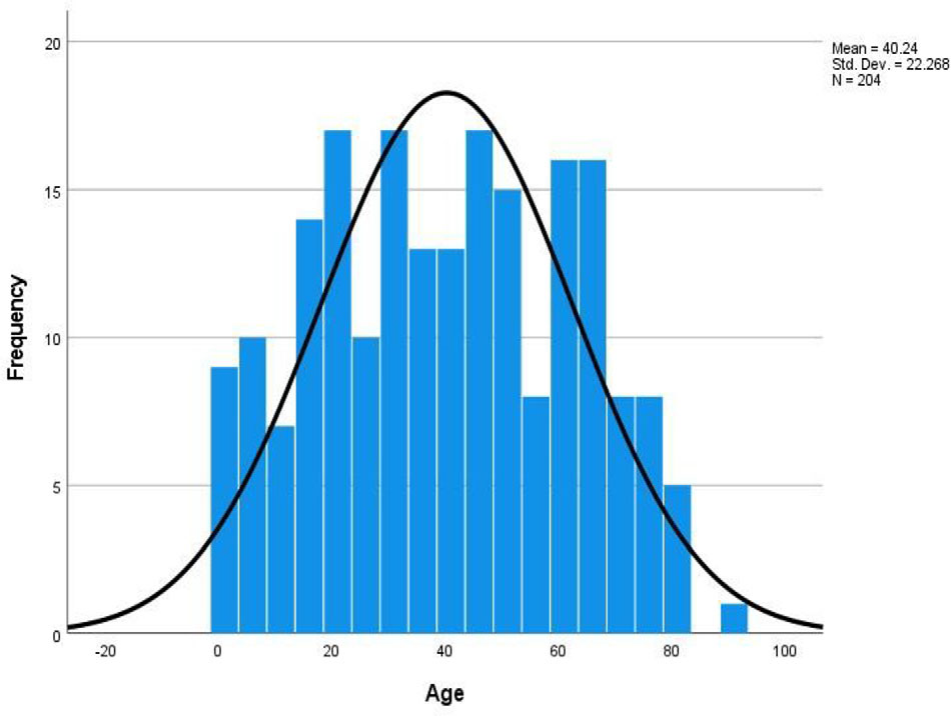
Age distribution of participants.

**FIGURE 2:**
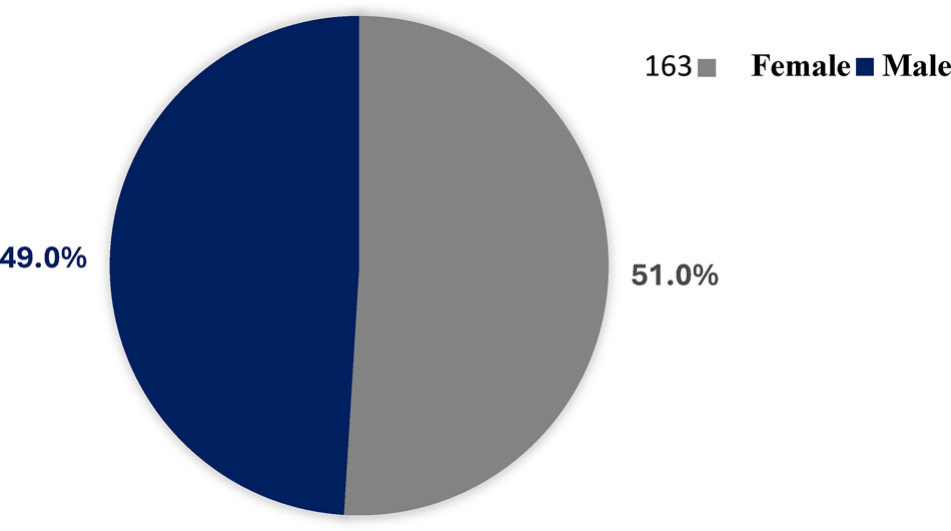
Gender distribution of participants.

The mean age at presentation was 40.24 ± 22.27 years, with a range of 1–90 years (Figure 1). The highest incidence of orofacial tumours was observed in the 5th decade of life, whereas the lowest incidence was observed in the 9th decade (Table 1). The study population comprised a slightly higher proportion of females (51.0%) than males (49.0%), with a male‐to‐female ratio of 1:1.04 (Figure 2).

**TABLE 1:** Age distribution by decade.

Decade	Frequency	Percentage
1st (1–10 years)	21	10.3
2nd (11–20 years)	23	11.3
3rd (21–30 years)	28	13.7
4th (31–40 years)	30	14.7
5th (41–50 years)	34	16.7
6th (51–60 years)	19	9.3
7th (61–70 years)	31	15.2
8th (71–80 years)	13	6.4
9th (81–90 years)	5	2.5
Total	204	100.0

### 3.2 Tumour Presentation Among Participants

More than half (59.8%) of the orofacial tumours identified in this study were benign in nature.

**FIGURE 3:**
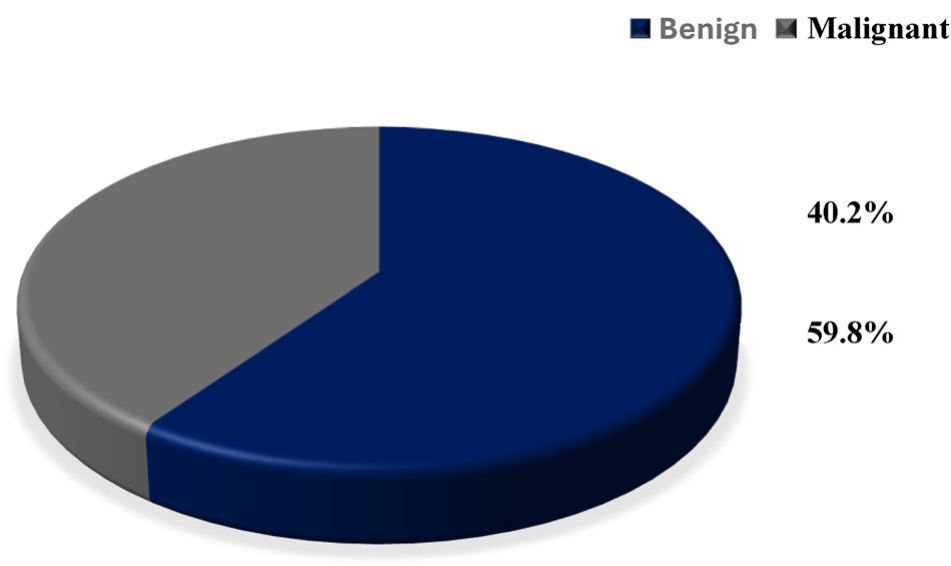
Types of tumours.

In contrast, two‐fifths (40.2%) of the cases involved malignant tumours (Figure 3).

**FIGURE 4:**
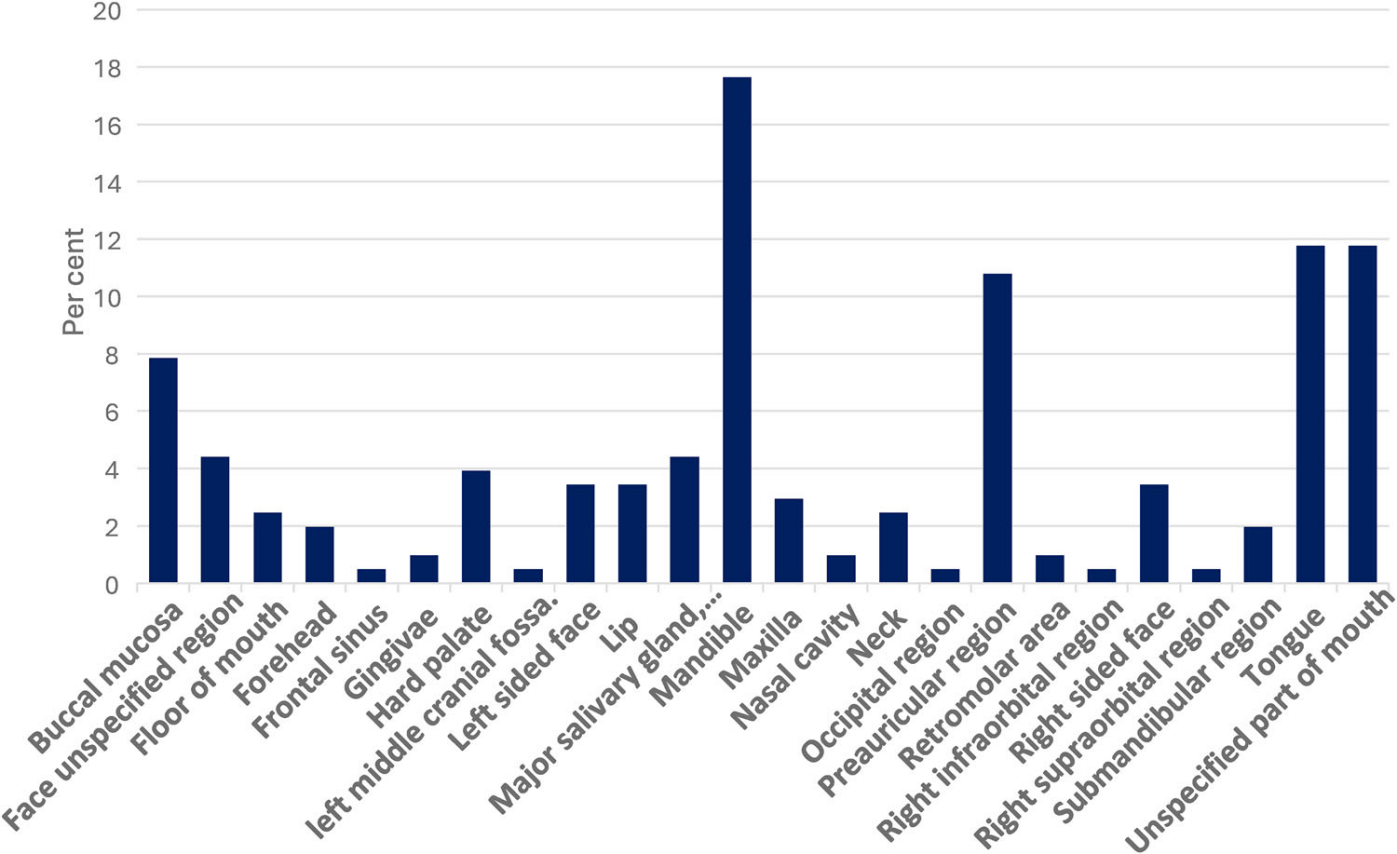
Tumour presentation.

Figure 4 displays the distribution of different sites affected by orofacial tumours in the study population. The site with the highest percentage of orofacial tumours was the mandible, accounting for approximately 18% of the cases. Other commonly affected sites included the tongue (∼12%), the mouth (∼12%) and the preauricular region (∼11%). Several other sites, such as the buccal mucosa, floor of the mouth, maxilla, lip, hard palate and neck, also exhibited notable percentages, ranging from around 2% to 8%.

**FIGURE 5:**
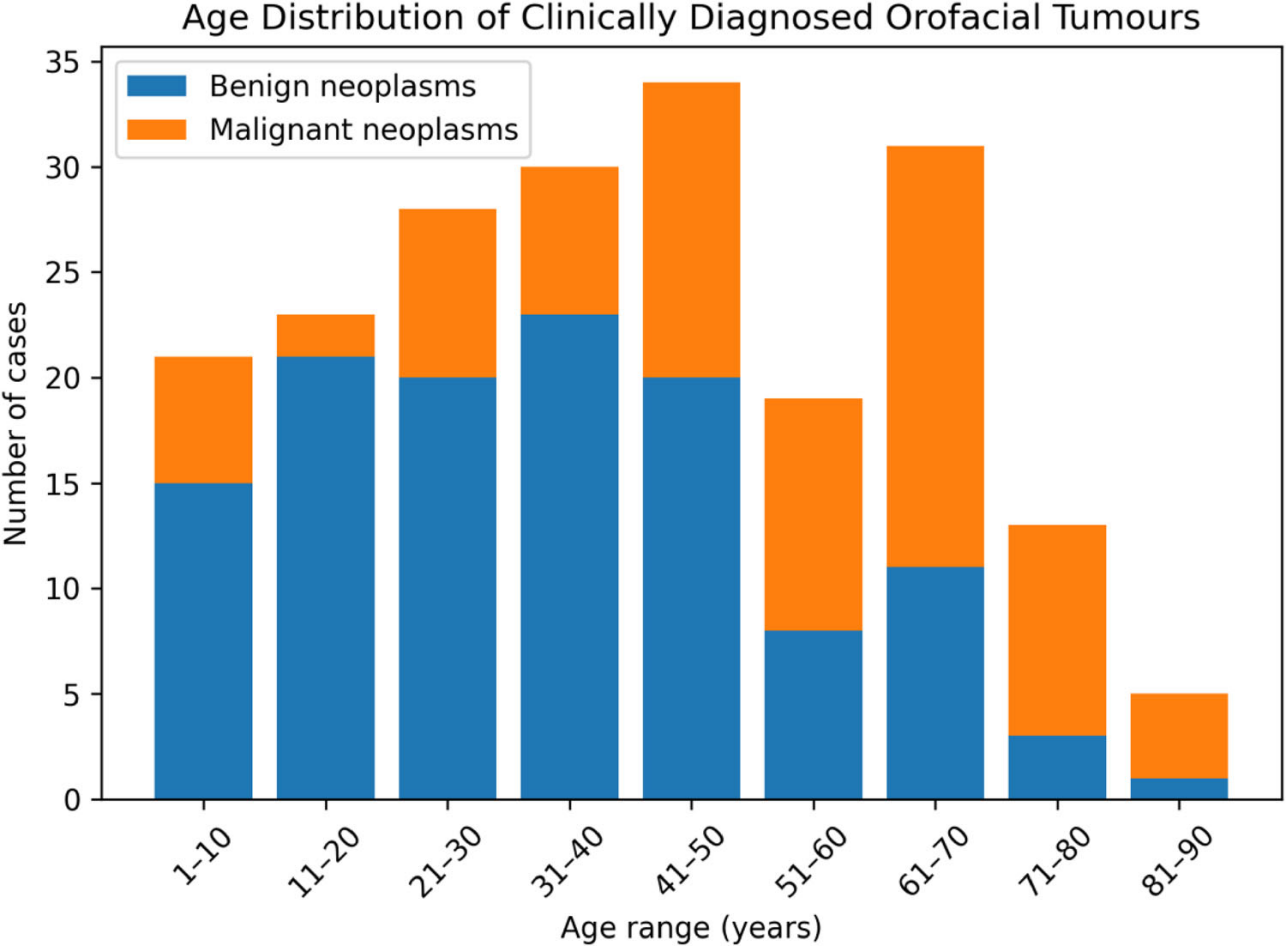
Age distribution of orofacial tumours among participants.

The age distribution of orofacial tumours (Figure 5) revealed distinct patterns for benign and malignant cases. Benign tumours were more prevalent in younger age groups, with the highest incidence observed in the 31–40 years age group (18.9%), followed closely by 11–20 years (17.2%) and 21–30 years (16.4%) age groups. In contrast, malignant tumours were more common in older individuals, with the 61–70 years age group exhibiting the highest incidence (24.4%). The second highest incidence of malignant tumours was noted in the 41–50 years age group (17.1%). Notably, the lowest incidence of benign tumours was found in the 81–90 years age group (0.8%), whereas malignant tumours were least common in the 11–20 years age group (2.4%). This age distribution suggests that the risk of developing malignant orofacial tumours increases with advancing age, whereas benign tumours tend to occur more frequently in younger individuals.

Regarding the gender distribution of orofacial tumours, a contrasting pattern emerged between benign and malignant cases (Table 2). For benign tumours, more than half (53.3%) of the cases were observed in females, whereas less than half (46.7%) occurred in males. Conversely, malignant orofacial tumours exhibited a slight predominance in males, with just over half of the cases occurring in the male population and the remaining cases affecting females.

**TABLE 2:** Gender distribution of orofacial tumours among participants.

	Tumour type			
	Benign		Malignant	
Gender	Count	Percentage	Count	Percentage
Female	65	53.3	39	47.6
Male	57	46.7	43	52.4

Table 3 presents a detailed breakdown of tumour site distribution for benign and malignant neoplasms in the orofacial region. For benign tumours, the mandible was the most common site (22.13%), followed by the preauricular region (17.21%) and unspecified parts of the mouth (13.93%). Malignant neoplasms showed a different pattern, with the tongue being the most frequent site (19.51%), followed by the buccal mucosa (12.20%) and the mandible (10.98%). Some sites showed marked differences in the prevalence of benign versus malignant tumours. The preauricular region had a high prevalence of benign tumours (17.21%) but a very low prevalence of malignant ones (1.22%). Conversely, the tongue showed a higher prevalence of malignant tumours (19.51%) compared to benign ones (6.56%). Certain sites were exclusively associated with either benign or malignant tumours in this cohort. Tumours in the neck and forehead were all benign, whereas those in the nasal cavity, retromolar area and various cranial regions were all malignant.

**TABLE 3:** Distribution of benign and malignant orofacial tumours by anatomical site.

Tumour site	Frequency	Percentage
**A: Distribution of benign orofacial tumours by anatomical site**		
Mandible	27	22.13
Preauricular region	21	17.21
Unspecified part of mouth	17	13.93
Tongue	8	6.56
Face (unspecified region)	8	6.56
Major salivary gland (unspecified)	7	5.74
Buccal mucosa	6	4.92
Neck	5	4.1
Right‐sided face	5	4.1
Forehead	4	3.28
Left‐sided face	4	3.28
Lip	3	2.46
Floor of mouth	3	2.46
Hard palate	2	1.64
Submandibular region	1	0.82
Maxilla	1	0.82
Total: 122 (100.0%)		
**B: Distribution of malignant orofacial tumours by anatomical site**		
Tongue	16	19.51
Buccal mucosa	10	12.2
Mandible	9	10.98
Unspecified part of mouth	7	8.54
Hard palate	6	7.32
Maxilla	5	6.1
Lip	4	4.88
Submandibular region	3	3.66
Left‐sided face	3	3.66
Gingivae	2	2.44
Nasal cavity	2	2.44
Retromolar area	2	2.44
Major salivary gland (unspecified)	2	2.44
Floor of mouth	2	2.44
Right‐sided face	2	2.44
Frontal sinus	1	1.22
Occipital region	1	1.22
Right infraorbital region	1	1.22
Right supraorbital region	1	1.22
Left middle cranial fossa	1	1.22
Total: 82 (100.0%)		

### 3.3 Associations Between Age, Gender and Tumour Type

A chi‐square test showed that there was a significant association (*p* < 0.001) between age and the type of tumour (Table 4). However, there was no significant association (*p* = 0.42) between gender and tumour type (Table 5).

**TABLE 4:** Association between age (decade) and tumour type.

	Tumour type			
Decade	Benign	Malignant	Total	*p* value
1st	15	6	21	
2nd	21	2	23	
3rd	20	8	28	
4th	23	7	30	
5th	20	14	34	<0.001*
6th	8	11	19	
7th	11	20	31	
8th	3	10	13	
9th	1	4	5	
Total	122	82	204	

* *p*<0.05.

**TABLE 5:** Association between gender and tumour type.

	Tumour type			
Gender	Benign	Malignant	Total	*p* value
Female	65	39	104	
Male	57	43	100	0.423
Total	122	82	204	

## 4 Discussions

### 4.1 Demographic Features

The mean age at presentation, 40.24 ± 22.3 years, observed in this study is consistent with findings from other studies in the West African sub‐region, though some variations exist. For instance, [11] reported a slightly higher mean age of 43 years for patients with orofacial tumours at the Korle‐Bu Teaching Hospital (KBTH) in Ghana. Conversely, [14] found a lower mean age of 30.5 ± 12.9 years in a Nigerian study. A 10‐year retrospective study by Kamulegeya and Kalyanyama [15] in East Africa revealed a mean age of 29.29 ± 19.7 years, which is lower than the findings of the present study.

The peak incidence of orofacial tumours was observed in the 5th decade of life, aligning with the findings of Gbotolorun et al. [16] in Lagos, Nigeria. However, other studies have reported different patterns; for example, Lawal et al. [17] found the highest incidence in the 3rd decade of life, whereas Dereje et al. [18] in Ethiopia reported the highest incidence in the 2nd decade. Although the 5th decade represents the peak incidence in this cohort, recent case reports emphasize that pleomorphic adenomas can present across a wide age demographic, including atypical presentations in both younger adults and the elderly [8]. This variability underscores the necessity for vigilance and thorough screening across a broad age spectrum. These variations highlight the influence of regional factors, study populations and methodologies on the observed age patterns of orofacial tumour presentation.

In terms of gender distribution, this study noted a slight female predominance (51.0%) compared to males (49.0%), resulting in a male‐to‐female ratio of 1:1.04. Although this distribution is similar to a study at KATH [19], it contrasts with findings from other African studies that generally reported a male predominance. For instance, Bassey et al. [14] documented a notable male predominance in Nigeria with a 2:1 ratio, whereas Parkins et al. [11] indicated a male predominance with a 1.2:1 ratio, and Dereje et al. [18] found a similar figure in Ethiopia. Although the slight female predominance found in this study is uncommon, it is not entirely unusual. Der et al. [20] observed a similar trend in northern Ghana, with a male‐to‐female ratio of 1:1.15, and Gbotolorun et al. [16] noted a more significant female dominance in Nigeria, with a 1:2.2 ratio. Differences in gender distribution across studies could stem from variations in sample sizes, populations examined and the types of orofacial tumours studied. These variations in gender distribution across different studies may be attributed to several factors. Differences in study populations, sample sizes and the types of orofacial tumours included in each study could contribute to the observed disparities.

## 5 Tumour Presentation Among Participants

### 5.1 Benign vs. Malignant Tumour Prevalence

This study found that benign tumours were more common (59.8%) than malignant ones (40.2%). This trend is consistent with recent research from West Africa and Ghana, although specific ratios differ. For instance, Okoh et al. [21] identified a much higher proportion of benign tumours (97.1%) compared to malignant (2.9%) in Nigeria. Similarly, Aregbesola et al. [22] found benign tumours represented 98.5% versus 1.5% malignant in another Nigerian study. In Ghana, Der et al. [20] reported values closer to this study, with 66.0% benign and 34.0% malignant tumours. However, it is important to note that not all studies in the region report a higher prevalence of benign tumours. Two notable exceptions are the studies by Kamulegeya and Kalyanyama [15] and Parkins et al. [11]. Kamulegeya and Kalyanyama reported a higher prevalence of malignant tumours (67.28%), whereas Parkins et al. found that 59% of orofacial tumours were malignant. The variations in tumour prevalence can result from differences in the populations studied, diagnostic criteria, access to healthcare and specific environmental or genetic risk factors in different regions. Clinically, the distinction between benign and malignant presentations can be challenging. Current literature notes that long‐standing or atypically located benign tumours may exhibit features that mimic malignancy, highlighting the critical importance of definitive histopathological confirmation and complete surgical excision to prevent recurrence [8]. Changes over time in exposure to various risk factors could also play a role.

### 5.2 Anatomical Site Distribution of Tumour

In this study, tumours frequently affected the mandible, tongue, preauricular region and buccal mucosa, a pattern that aligns with findings from West Africa and other African nations. Bassey et al. [14] determined that the mandible was the primary site for oral and maxillofacial tumours in Nigeria. Similarly, Dereje et al. [18] in Ethiopia identified the mandible, maxilla and buccal mucosa as common locations for orofacial tumours. Leigh et al. [23] in Gambia also reported the mandible as the most frequent site. These areas are particularly vulnerable to tumour development due to exposure to various carcinogenic agents arising from habits such as tobacco use, alcohol consumption and viral infections [24].

### 5.3 Age Distribution

The study indicates that benign tumours were more likely to be found in younger age groups, especially those aged 31–40 years (18.9%), followed closely by the 11–20 years (17.2%) and 21–30 years (16.4%) age groups. In contrast, malignant tumours were predominantly seen in older patients, particularly in the 61–70 years age group, which exhibited the highest incidence (24.4%). The distinct patterns in age distribution for benign and malignant tumours corroborate existing literature from Africa and other regions.

A study by Aregbesola et al. [22] in Nigeria reported that benign tumours were more prevalent in younger age groups (31.44 ± 13.64 years), whereas malignant tumours were more common in older individuals, consistent with the findings of the present study. Furthermore, a study by Bassey et al. [14] found that the incidence of oral and maxillofacial malignancies increased with advancing age, similar to the observed peak incidence of malignant tumours in the 61–70 years age group in the current study.

A chi‐square test demonstrated a significant association between age and tumour type (*p* < 0.001, Table 4), suggesting that the occurrence of various types of orofacial tumours varies significantly across different age groups. This aligns with a study by Joseph et al. [25] in Kuwait. The age‐specific patterns may be attributed to factors such as cumulative exposure to carcinogenic agents, age‐related immune system changes and genetic alterations that accumulate over time [26].

### 5.4 Gender Distribution of Tumours

A higher prevalence of benign tumours was observed in females (53.3%), whereas malignant tumours were more common in males (52.4%). A study by Parkins et al. [11] in Ghana reported a higher incidence of malignant tumours in males; however, evenly distributed benign tumours among males and females. Another study by Bassey et al. [14] in Nigeria found a higher prevalence of both benign and malignant tumours among females. However, it is important to note that a chi‐square test showed no significant association between gender and type of orofacial tumour (*p* = 0.423, Table 5).

The divergent gender distribution patterns for benign and malignant tumours observed in this study may be influenced by various factors, including differences in risk factor exposure (e.g., tobacco and alcohol use), hormonal influences and health‐seeking behaviours between males and females [24]. Further research is needed to investigate the underlying reasons for these gender‐specific patterns in the Ghanaian population and identify potential gender‐specific risk factors. Referral bias also could have accounted for differences in gender distribution.

## 6 Conclusion

This 2‐year retrospective study conducted at the Oral and Maxillofacial Unit of KATH has shed light on the patterns and clinical characteristics of orofacial tumours in the Ghanaian population. The results indicated a slight predominance of tumours in females, with benign tumours occurring more frequently than malignant ones. This gender distribution contrasts with findings from other studies in the region, suggesting that there may be demographic differences in tumour presentations across various West African populations.

The mandible was identified as the most common site for tumour occurrence, followed by the tongue and preauricular region. This anatomical distribution aligns with findings from other studies in Africa and emphasizes the importance of thorough examination of these sites during routine oral health check‐ups. A significant association was observed between age and tumour type, with benign tumours more prevalent in younger age groups and malignant tumours more common in older individuals. This age‐related trend points to the importance of implementing age‐specific screening protocols and raises questions about the long‐term effects of various risk factors. Although there were differences in gender distribution between benign and malignant tumours, no significant link was established between gender and tumour type, indicating that other factors may be more influential in determining tumour malignancy.

Furthermore, this study provides a foundation for future longitudinal research to track changes in orofacial tumour patterns over time in Ghana. It also highlights the importance of maintaining comprehensive patient records and the potential value of establishing a national tumour registry. The insights gained from this study can inform public health strategies, guide resource allocation in oral healthcare and contribute to the development of targeted prevention and early intervention programmes.

In conclusion, although this study has explored the epidemiological characteristics of orofacial tumours in Ghana, it also illustrates the complexity of these neoplasms and the need for ongoing research. As further studies explore the intricacies of orofacial tumour development and progression, healthcare practices can evolve towards more effective prevention, early detection and treatment strategies, ultimately enhancing patient outcomes and quality of life.

## 7 Recommendations

To improve the understanding, diagnosis and management of orofacial tumours in Ghana, several key strategies are recommended. First, targeted public health campaigns should be developed to raise awareness of orofacial tumours, their risk factors and the importance of early detection. These campaigns can help promote timely health‐seeking behaviour and improve outcomes through early intervention. Additionally, training programmes for healthcare professionals should be enhanced to build capacity in the early diagnosis and effective management of these conditions. This includes equipping general practitioners, dentists and primary care workers with the knowledge and tools needed to recognize early signs and ensure prompt referrals.

Another crucial step is the establishment of a national registry for orofacial tumours. This registry would facilitate long‐term epidemiological studies, enable the monitoring of disease trends and provide valuable data to support healthcare planning and policy formulation. To optimize patient care, there should also be strengthened multidisciplinary collaboration between oral and maxillofacial surgeons, oncologists, pathologists and other relevant specialists. This collaborative approach would enhance treatment planning, improve clinical outcomes and ensure comprehensive care for affected patients.

Furthermore, this study provides a foundation for future longitudinal research to track changes in orofacial tumour patterns over time in Ghana. It also highlights the importance of maintaining comprehensive patient records and the potential value of establishing a national tumour registry. Improving documentation practices is vital. This can be achieved by training clinical staff on accurate and complete record‐keeping, as well as developing and implementing a user‐friendly electronic medical record (EMR) system tailored specifically for orofacial tumour cases. Finally, further research should be conducted to investigate environmental, genetic and lifestyle factors that may contribute to the development of orofacial tumours in the Ghanaian population. Such research could provide deeper insights into the aetiology of these tumours and inform effective prevention strategies.

## Author Contributions

Gift Obiageri Maduagwu and Nana Bempong Owusu‐Ankomah contributed to the concept, visualization, data collection and manuscript reviews. Nana Atuahene Oti, Afia Boko Ampe Asare and John Billy Owusu Quarshie contributed to the data collection, formal analysis and manuscript review. Elijah Kwegyir Johnson and Kwame Adu Okyere Boadu contributed to the methodology, resources and manuscript's original write‐up and review.

## Funding

The authors have nothing to report.

## Disclosure

All authors have read and approved the final version of the manuscript. Kwame Adu Okyere Boadu had full access to all the data in this study and takes complete responsibility for the integrity of the data and the accuracy of the data analysis.

## Conflicts of Interest

The authors declare no conflicts of interest.

## Transparency Statement

Kwame Adu Okyere Boadu affirms that this manuscript is an honest, accurate and transparent account of the study being reported; that no important aspects of the study have been omitted; and that any discrepancies from the study as planned have been explained.

## Data Availability

The authors confirm that the data supporting the findings of this study are available within the article.
